# High Rates of Potentially Infectious Tuberculosis and Multidrug-Resistant Tuberculosis (MDR-TB) among Hospital Inpatients in KwaZulu Natal, South Africa Indicate Risk of Nosocomial Transmission

**DOI:** 10.1371/journal.pone.0090868

**Published:** 2014-03-13

**Authors:** Nonkqubela Bantubani, Gaetan Kabera, Catherine Connolly, Roxana Rustomjee, Tarylee Reddy, Ted Cohen, Alexander S. Pym

**Affiliations:** 1 TBRU, Medical Research Council, Durban, KwaZulu-Natal, South Africa; 2 Harvard Medical School Division of Global Health Equity, Brigham and Women's Hospital, Boston, Massachusetts, United States of America; 3 KwaZulu-Natal Research Institute for Tuberculosis and HIV Research (K-RITH), Nelson Mandela School of Medicine, Durban, KwaZulu-Natal, South Africa; McGill University, Canada

## Abstract

**Background:**

Nosocomial transmission has been implicated as a key factor in the outbreak of extensively drug resistant (XDR) and multidrug-resistant (MDR-TB) tuberculosis at Church of Scotland Hospital (CoSH), in KwaZulu-Natal (KZN), South Africa. The aim of this study was to quantify the burden of potentially infectious tuberculosis and the proportion of drug resistance among hospital inpatients throughout the province of KZN.

**Methods:**

Inpatients with current cough, capable of producing sputum were selected from 19 public hospitals in KZN. After informed consent, demographic and clinical data, and sputum samples were collected. Samples were processed for fluorescent microscopy, liquid culture and first and second-line anti-tuberculosis drug susceptibility testing.

**Results:**

There were a total of 2,964 inpatients where sampling was done. About 1,585 inpatients (53%) had a current cough and sufficient microbiological and clinical data for inclusion. *Mycobacterium tuberculosis* was isolated from 543 inpatients (34% of those tested and 18% of all inpatients). Eighty-four (15%) inpatients with TB were found to be MDR-TB infected and 16 (3%) had XDR-TB. There was no association between the prevalence of MDR-TB and proximity to CoSH. Among patients with microbiologically confirmed TB, MDR/XDR-TB was associated with male sex, a longer length of stay between hospital admission and date of sample collection, and current or previous TB treatment.

**Conclusions:**

One in five inpatients had potentially infectious TB. This is an underestimate since patients without current cough were not tested. MDR-TB was frequently observed and was found in nearly one in six active TB inpatients. While present at lower levels than the original outbreak report at CoSH, XDR-TB was detected in hospitals throughout KZN. The high burden of potentially infectious TB and confirmed MDR-TB, much of it undiagnosed, indicates a serious risk for nosocomial transmission and the need for intensified infection control within the inpatient setting.

## Introduction

HIV has had a huge impact on the incidence of tuberculosis (TB) cases in sub-Saharan Africa [1]. South Africa has an estimated adult HIV prevalence of 17.9% in 2012 [2] andthe estimated incidence of TB has risen from 317 to 1000/100,000 population between 1995 and 2012 [3]. In 2012 65% of HIV tested tuberculosis cases were co-infected with HIV [3] and together these two pathogens are responsible for an estimated 46% of disability-adjusted life years (DALY) lost in South Africa [1]. While the HIV epidemic alone has complicated local responses and increased resource demands on an already strained public health system, the spread of drug-resistant TB (DR-TB) within South Africa constitutes an additional challenge to effective control of the disease.

Based on data from the most recent national Drug Resistance Survey in 2002, South Africa had an estimated burden of approximately 13,000 multidrug-resistant TB (MDR-TB) cases [4]. MDR-TB is defined as resistant to isoniazid and rifampin. This MDR-TB burden places South Africa on the 5^th^ position among countries with the highest global incidence of MDR-TB after China, India, Russian Federation, and Pakistan in terms of absolute numbers [5]. The top four countries have an estimated HIV prevalence of less than 1% compared to South Africa where the HIV prevalence is estimated to be 17.9% [2]. The urgency of addressing DR-TB emergence in a high HIV prevalence setting was underscored by the report of an outbreak of extensively drug-resistant TB (XDR-TB) in Tugela Ferry centered in and around the Church of Scotland Hospital (CoSH) in the Msinga sub-district of KwaZulu-Natal (KZN) [6]. XDR-TB is a form of MDR-TB that is additionally resistant to a fluoroquinolone and either amikacin, kanamycin or capreomycin and is associated with a high mortality [6]. Subsequent analysis identified evidence for a substantial role of nosocomial transmission in this highly publicized XDR-TB outbreak [7], [8].

The role of healthcare institutions in the propagation of TB, and highly-drug resistant tuberculosis, in South Africa is not clear. While the Tugela Ferry outbreak provides a concrete example of how the concentration of immuno-suppressed patients on open wards can facilitate the spread of tuberculosis and DR-TB in particular, the more general role of nosocomial transmission in the transmission and amplification of DR-TB requires further study. Does CoSH represent a unique infection control phenomenon, or does it represent a more general problem in KZN? Previous studies have documented a large burden of potentially infectious TB among inpatients [9] and undiagnosed TB among patients dying at Edendale Hospital, located in the Msunduzi sub-district of KZN [10].

In this study we aim to quantify the burden of potentially infectious TB within hospital settings throughout the KZN Province, to estimate the proportion of inpatients with DR-TB, and to identify risk factors for drug resistance among these potential sources of nosocomial TB transmission.

## Methods

### Study Setting

KZN is the second most populated province in South Africa with a population of over 10 million, just over 20% of the total South African overall population. In 2012, the incidence of TB in South Africa was 1,000/100,000 with the highest burden of tuberculosis in KZN [3]. The HIV prevalence among antenatal clinic attendees is over 37.4%[11].

**Figure 1:**
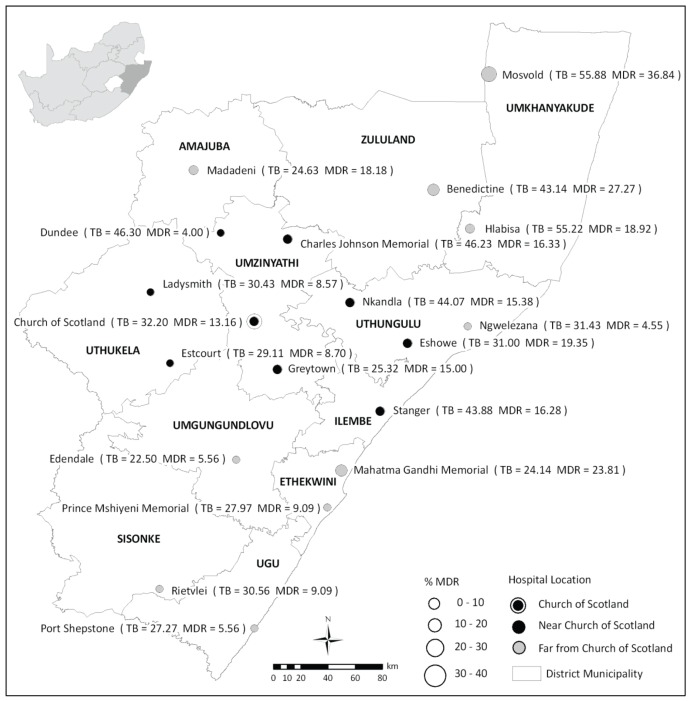
Proportion of Symptomatic Inpatients with Culture confirmed Tuberculosis and MDR-TB by hospital and District of KwaZulu-Natal. Patients were defined as symptomatic if they were coughing on the day of sampling. The diameter of the circles representing hospitals is proportional to the proportion of all culture positive patients with MDR-TB. The shading of the circles shows whether the hospitals were adjacent to Church of Scotland Hospital (CoSH) at Tugela Ferry. The figures in brackets at each hospital indicate the proportion (%) of all sampled patients who were culture positive and the proportion who had confirmed MDR-TB.

KZN is divided into 11 health districts and has 74 public hospitals. It was not feasible to include all public hospitals in this study. The study was, therefore, restricted to larger public hospitals (>200 inpatient beds) that admitted general medical patients. TB or referral/specialist hospitals were excluded. Thirty-eight hospitals met these broad inclusion criteria. Given logistical and resource constraints, we further reduced the number of hospitals to be included in our study. In order to describe possible local extension of XDR-TB, the nine eligible hospitals in closest proximity to Church of Scotland were selected and, in order to examine the burden of resistance in the rest of the province, an additional nine hospitals were randomly selected from the rest of the study area (Figure 1). Each of these 18 hospitals was sampled at two separate time points which allowed us to test for temporal changes in the proportion of disease that was resistant within these settings (Phase 1: August 2007 to August 2008 and Phase 2: August 2009 to November 2009). One additional hospital (Benedictine) was included only in Phase 2 of the study.

### Study Population

Participant enrolment was conducted at each selected facility on a single day during each of the two phases of the study. Adult inpatients (at least 18 years old) on medical wards, TB wards, isolation wards and in some cases from surgical wards (where patients had been moved due to scarcity of beds in medical wards) were screened for inclusion in the study. Any patient reporting a cough on the day of the survey (regardless of other conditions or the reason for admission to hospital) and who could provide informed consent was enrolled. Thus the study population consisted of inpatients with unsuspected TB, suspected TB and diagnosed TB. Participants were sampled regardless of whether they had initiated TB chemotherapy. After written informed consent was obtained, using a consent form approved by the Biomedical Research Committee of the University of KwaZulu-Natal, participants were given containers to collect sputum in the early hours of the following day. For participants with an unproductive cough, the sputum was induced with hypertonic saline. The study team collected samples following the local infection control guidelines [12]. A standardized questionnaire including demographic and clinical data was also administered by trained field staff. Patients' hospital records were reviewed for date of admission, diagnosis, current treatment, microscopy results and verification of HIV status.

Most of the selected hospitals lack respiratory isolation facilities and the vast majority of the wards from which participants were enrolled were open, shared space. Patients diagnosed with TB during the course of this study were started on treatment and transferred to TB-specific wards within these hospitals when available, consistent with standard local practice.

### TB culture and drug-susceptibility testing

Sputum samples were processed using N-acetyl-L-cysteine-sodium hydroxide [13] and centrifugally concentrated for acid fast bacilli smear examination and *M. tuberculosis* culture. The concentrated pellet was suspended in 1.25 mls of buffer (pH 6.8) following which 0.1 ml was placed on a microscope slide and 0.5 ml was cultured on both Middlebrook 7H11 selective agar (Difco) and liquid medium (BACTEC MGIT 960, Becton Dickenson Diagnostics). Positive cultures were confirmed as *M. tuberculosis* using Niacin and Nitrate biochemical testing [13]. A Middlebrook 7H10 agar (Difco), 1% proportion test of susceptibility to isoniazid (H = 1 ug/ml), rifampicin (R = 1 ug/ml), ethambutol(E = 7.5 ug/ml), streptomycin(S = 2 ug/ml), kanamycin (K = 10 ug/ml), ofloxacin (O = 1 ug/ml), ethionomide (ETH = 5.0 ug/ml), capreomycin (C = 5 ug/ml) was performed on all *M. tuberculosis* confirmed cultures.

Specimens were classified as confirmed *M. tuberculosis* if either 7H11or MGIT was positive. Patients resistant to H and R (with or without resistance to additional anti-tuberculosis drugs) were classified as MDR-TB infected. Patients with MDR-TB and additional resistance to O and the injectable K or C were classified as XDR-TB infected. The prevalence rate of MDR- and XDR-TB was calculated as a proportion of confirmed cases of TB.

### Statistical methods

Data was double entered into EpiData (Odense, Denmark) from paper records and statistical analyses were done using Stata version 11.0 (College Station, TX, USA). Data was analysed using commands in Stata Version 11.0 (College Station, TX, USA) software which takes into account the cluster sampling design. We first described the demographic and clinical characteristics of study participants. We then reviewed the bacteriological results and used a chi square test, adjusted for clustering by hospital, to test for differences in the proportion of TB that was resistant by proximity to CoSH and by Phase of the study. Finally, we identified factors independently associated with MDR-TB among those with detected TB using multivariate logistic regression. Our multivariate model initially included all factors. Factors were eliminated one by one starting with the least significant until all factors remaining were significant (p-value<0.05).

### Ethics Statement

The study received ethics approval from the University of KwaZulu-Natal Biomedical Research Ethics Committee (BREC). A patient information sheet and informed consent document was approved by BREC for use in the study.

## Results

There were a total of 2964 inpatients on the hospital wards on the sampling days that were included in our study. We consented and attempted to collect sputum specimens and clinical data from a total of 1644 symptomatic inpatients at participating hospitals (758 during Phase 1 and 886 during Phase 2). Fifty-nine potential participants for whom we were missing clinical or microbiological data were excluded from the analyses (57 during Phase 1 and 2 during Phase 2). In total, our analysis included data from 1,585 inpatients (which represented 53% of all inpatients).

### Characteristics of the sample population

Most measured characteristics (sex, age, on TB treatment, TB suspected, diagnosed with HIV) were similar among participants in the two Phases of the study (Table 1).

**Table 1:** Demographic and clinical characteristics of study population.

Characteristics	n	%
**Participants**	1585	100
**Age**, (median, IQR)	1544	37(30–48)
**Sex (male)**	796	50%
**On TB Treatment (yes)**	811	51%
**Cough**		
No cough	158	10%
<2 wks	308	19%
2–3 weeks	409	26%
4 or more	591	37%
Unknown	119	8%
**TB Close contact (yes)**	601	42%
**Previous treatment (yes)**	584	37%
**HIV status**		
Pos	868	55%
Neg	263	17%
Unknown/refused	454	29%
**>1 Hospital admission <2 yrs**	509	32%
**>4 Outpatient visits <2 yrs**	817	52%

The proportion of participants on treatment for TB at the time of the survey was similar in both phases (51%). Of those on treatment, 20% had initiated treatment before the current admission, 31% at the time of admission and 48% at some point between admission and time of data collection. A higher proportion of participants had started treatment prior to admission in the second Phase of our study compared with the first (24% vs 15%, p = 0.02). Of those on treatment for TB, most were taking a standard first line fixed dose combination of RHZE or RH (83%) and 21% were being treated with streptomycin.

### Detection of *M. tuberculosis* among study participants

A case of tuberculosis was defined on the basis of the ability to grow *M. tuberculosis* from sputum using either solid or liquid media. From the 1,585 inpatients included in the analysis 543 (34%) (Table 2) were found to be *M. tuberculosis* culture positive cases. The yields from both phases were similiar (36% in Phase 1 and 33% in Phase 2). Auramine smear results were available for 542 of the culture positive patients, and 317 (58%) of these were auramine smear positive. In addition there were 59 patients who were found to be smear positive but had negative cultures with both culture media. The majority of these smear positive culture negative patients, 39 (66%), were on tuberculosis therapy at the time of sampling which could account for the discordance between the two tests. Of participants not receiving TB treatment at the time of sampling, a total of 26% were bacteriologically confirmed TB. Of patients on treatment at the time of the survey, 42% still had positive cultures and thus remained potential sources of nosocomial transmission.

**Table 2:** Microbiology results for two phases of the study for all sampled inpatients.

Microbiology	N^a^	%^b^
**Auramine Smear**		
Positive	376	24%
Negative	1206	76%
**Culture^c^**		
Positive	543	34%
Other	1042	66%
**MDR^d^**	84	15%
**XDR**	16	2%

The symbols N^a^ and %^b^ correspond to the total number and percentage of sampled patients with the indicated result. Culture^c^ is a composite result of MGIT and 7H11 agar. Culture Positive refers to growth of *M. tuberculosis* on either media, and culture other to negative on both media or no result. MDR^d^ is resitance to both rifampicin and isoniazid and is inclusive of XDR.

### Multidrug resistance

Of the 543 positive MTB isolates, 472 (87%) had drug-susceptibility results. Of the 71 without susceptibility results, 25 were contaminated, 27 did not regrow during drug susceptibility testing, and 19 were not tested. Of 543 culture confirmed cases of *M tuberculosis*, 84 specimens (15%; 95CI: 12%–20%) were identified with MDR-TB (inclusive of XDR-TB). The proportion of MDR-TB amongst culture positive patients was similar in patients attending CoSH (13%), the eight facilities closest to CoSH (13%) and the ten facilities further from COSH (18%), p = 0.1. In total, 16 cases of XDR-TB were detected (3%; 95 CI: 2%–5%). There was no difference in the proportion of XDR-TB among the three geographic groups of facilities (p = 0.1) [Table 3]. However, the proportion of MDR-TB at individual facilities varied considerably from a high of 37% in Mosvold to a low of 4% in Dundee (Figure 1).

**Table 3:** Proportion of Culture Positive and MDR-TB Inpatients by hospital proximity to Church of Scotland Hospital.

	Total study population						
	Specimens	Culture positive		MDRb		XDR	
Hospital location	n	n	%	n	%	n	%
CoSHa	118	38	32%	5	13%	3	8%
Adjacent to CoSH	690	252	37%	34	13%	7	3%
Not adjacent to CoSH	777	253	33%	45	18%	6	2%
Total	1585	543	34%	84	15%	16	3%

CoSHa refers to Church of Scotland Hospital at Tugela Ferry. Adjacent to CoSH corresponds to 8 hospitals in sub-districts neighbouring CoSH. Not adjacent to CoSH corresponds to 10 hospitals at locations not neighbouring CoSH. Culture positive is the number and percentage of sampled individuals with culture positive tuberculosis.

MDRb is the number and percentage of sampled individuals with rifampicin and isoniazid resistance including XDR-TB.

Amongst the 472 participants with culture positive samples and available susceptibility testing, 104 had resistance to any TB drug (22%). Resistance to individual anti-TB drugs were as follows: H = 96 (20.3%); R = 88 (18.6%); E = 68 (14.4%); S = 64 (13.6%); ETH = 33 (7.0%); O = 20 (4.2%); K = 17 (3.6%) and C = 14 (3.0%).

The proportion of cases with MDR-TB differed between newly diagnosed patients (10%) and those previously treated for TB (32%), p<0.001. The majority of MDR-TB patients were on some form of TB treatment (85%) [Table 4]. The levels of mono-resistance and poly-resistance to first line drugs were similar in new and previously treated cases.

**Table 4:** Anti TB drug resistance profile of TB patients with Drug Susceptibility results.

	New case^a^						Previous Case					
	On Treatment						On Treatment					
Resistance	Yes	%	No	%	Total	%	Yes	%	No	%	Total	%
Pan Sensitive	137	84	107	91	244	85	79	60	28	68	107	62
Monoresistent	2	1	2	2	4	1	2	2	4	10	6	3
Polyresistent(not MDR)	2	1	2	2	4	1	3	2	2	5	5	3
MDR+XDR	23	14	6	5	29	10	48	36	7	17	55	32
XDR	5	3	2	2	7	2	9	7	0	0	9	5
Total	164	100	117	100	288	100	132	100	41	100	173	100

a 11 patients had unknown previous treatment.

### Factors independently associated with MDR among those with TB

In a multivariate logistic regression model that adjusted for the cluster study design, male sex [OR_adj_: 2.0; 95% CI: 1.4–3.1], a longer time between hospital admission and date of sample collection [OR_adj_: 1.1; 95% CI: 1.0–1.2] (a 10% increased odds of MDR for every additional day of hospitalization), being currently on TB treatment [OR_adj_: 2.5; 95%CI:1.4–4.5] and being treated for TB before admission [OR_adj_: 3.5; 95%CI: 1.8–6.71] were independently associated with MDR among those with TB (Table 5).

**Table 5:** Analysis of factors associated with MDR.

		Univariate results		Multiple logisitic results	
Variable	n	OR (95% CI)	p value	OR (95% CI)	p value
Phase (ref = phase 1)	543	0.94 (0.55–1.60)	0.8		
Sex (ref = male)	542	1.57 (1.05–2.36)	0.03	2.01 (1.41–3.13)	0.001
Age in increments of 5 years	527	0.95 (0.84–1.08)	0.42		
Length of stay from Hospital admission to date of sample collection in increments of one week	536	1.14 (1.03–1.27)	0.01	1.10 (1.00–1.20)	0.045
Currently on TB treat (ref = yes)	543	3.72 (2.16–6.42)	<0.001	2.52 (1.41–4.51)	0.004
Cough (ref = yes)	534	1.39 (0.54–3.52)	0.47		
Duration of cough in increment of one month	487	1.04 (1.02–1.06)	<0.001		
Treated for TB before admission (ref = yes)	522	3.95 (2.08–7.51)	<0.001	3.46 (1.78–6.73)	0.001
Last TB treatment completed (ref = No)	180	2.19 (0.96–4.99)	0.06		
Last TB outcome (ref = not cured)	174	3.16 (1.40–7.14)	0.01		
TB close contact (ref = Yes)	475	2.01 (1.24–3.25)	0.01		
Number of times admitted to an hospital >1 (ref = 1 time)	543	1.19 (0.66–2.14)	0.5		
Number of visits to any clinic or OP dep. >4 (ref = 1–4 times)	543	1.53 (0.92–2.54)	0.1		
Study Auramine Smear	542	1.68 (0.92–3.06)	0.09		

### Unrecognized and ineffectively treated tuberculosis in the hospital setting

There were 199 patients with bacteriologically confirmed TB that were not on TB treatment at the time of the survey. These patients had already had a median length of stay in hospital of 3 days (IQR: 2–6) prior to the survey. Fourteen participants had been in hospital for over 2 weeks at the time of the survey and 39% reported a cough lasting 4 weeks or more. We isolated viable *M. tuberculosis* from 344 patients that were on TB treatment at the time of the survey. These patients already had a median length of stay of 9 days in hospital (IQR: 5–19) and 49% reported having a cough for 4 weeks or more. This corresponded to a median duration of treatment of 7 days (IQR: 4–21) at the time of sampling.

Of the 84 patients categorized as MDR-TB by drug susceptibility testing conducted during the survey, only 8 were known to the health facility to be MDR-TB patients at the time of the sampling (10%). The remaining 76 participants with potentially infectious MDR-TB (90%) had already spent a median of 7 days (IQR: 4–22) in hospital with unrecognized MDR-TB. Of these, 14 participants had been in hospital for over a month. Almost half reported a cough lasting four weeks or more.

## Discussion

Outbreaks of TB and DR-TB within hospital settings have been documented in diverse geographical settings [14]–[17]. In South Africa, as in other settings where HIV infection is common among inpatients and the background prevalence of TB is high, the risk of nosocomial transmission of TB is likely to be amplified. As demonstrated at CoSH, when immuno-suppressed patients on open wards are exposed to other patients with unrecognized, or inadequately treated, highly drug resistant strains of *M. tuberculosis*, it is possible for large, highly lethal outbreaks to occur. Using a mathematical model, Basu predicted that the number of XDR-TB cases could increase five-fold in the absence of any intervention and that more than half were likely to be nosocomial [8]. The model also showed that clusters of initially nosocomial XDR-TB could extend into the community [7]. While the tragic outbreak and previous studies showing a high burden of TB among hospital inpatients in KZN [9], [10], it was not clear, at the time of initiating this study whether the threat of nosocomial transmission of highly drug resistant TB was generalized throughout the province.

In our survey, we found that nearly one in five inpatients (317) on wards visited for this survey were smear and culture-positive TB and posed a potential risk of transmitting disease within the hospital. An additional 20 patients, not on therapy at the time of sampling were smear positive but culture negative possibility due to loss of viability during transit of the sample from the peripheral hospitals. Since only patients with an active cough on the day of the survey were sampled, this may actually underestimate the burden of active disease on these wards. Out of 543 of all patients with microbiological evidence of active TB, 344 (63%) were already on treatment at the time of sampling for this survey. From these participants receiving TB treatment, it was still possible to detect *M. tuberculosis* from 42%. One possible reason for persistent smear or culture positivity despite treatment is that these patients had recently started their course of therapy and it was still too early to detect any smear or culture conversion.

A second possibility for the persistence of smear or culture positivity despite treatment is that these participants had DR-TB that was not likely to respond to first-line treatment. It is concerning that 90% of these patients with MDR-TB were not known to be infected with MDR-TB strains and could not have been treated with appropriate therapy. The multivariate model revealed strong associations between DR-TB and current or recent treatment for TB among those participants in whom we found viable *M. tuberculosis* at the time of survey; this is not unexpected as treated TB cases without MDR-TB would likely have responded and been culture negative at the time of the survey. The finding that longer hospital stays were also associated with MDR-TB may reflect persistent illness due to this resistant TB despite first-line treatment and demonstrates that these patients with MDR-TB may remain potential index cases in the wards for longer periods of time than those with drug susceptible TB.

Our data suggest that the risk of active MDR-TB among inpatients is a generalized threat throughout KZN and is not confined to CoSH or to the hospitals in the areas in immediate proximity to CoSH. While the proportion of MDR-TB varied substantially between facilities and appears to be highest in the northern health districts within KZN, we detected cases of active, potentially infectious MDR-TB at each facility visited. In contrast to the severity of MDR-TB throughout hospitals in KZN, consistent with previous reports [18], XDR-TB appears to be mainly concentrated in and around CoSH. We, however, found a small percentage of cases with XDR-TB even at facilities far away from the apparent epicenter of this outbreak. We note that these cross-sectional surveys were done at each hospital on a single day and that because of the high rates of patient turnover, this may not reflect the prevalence of TB or DR-TB among inpatients over longer periods of time. However the study was carried out in two phases with consistent results during each phase indicating the rates are likely to have been maintained during the study period

This study reveals a critical TB infection control challenge in hospitals throughout KZN. The high frequency of microbiologically confirmed potentially infectious TB among inpatients, both with drug susceptible and drug resistant forms of the disease, poses a serious threat for patients and hospital workers in this province [18]. The extent of the risk is hard to quantify exactly. Recent studies attempting to measure aerosol production have found considerable variability in culturable cough aerosols, even amongst smear positive patients [19], and aerosol production does correlate with recent transmission to household contacts [20]. It is also unclear how quickly patients become non infectious after initiation of therapy and 399 of the culture positive patients in this study had been on treatment for a median of seven days. Early studies suggested the infectiousness of patients with drug susceptible tuberculosis declined dramatically within 2 weeks of initiating therapy [21], but others have suggested that many smear positive patients remain infectious for much longer [22]. However these studies were carried out largely in the household contact setting and the parameters determining transmission in an open ward, between highly immune-compromised individuals have not be determined but could include lower bacillary sputum burdens.

The WHO has stressed the importance of infection control in strategies to improve TB control [23], but there are many unanswered questions about how to implement an effective infection control plan in an area with a disease burden as great as in KZN. Particular challenges raised by our data include the fact that nearly one in five participants in our study with a negative sputum smear was eventually diagnosed with TB based on culture. This suggests that the use of standard smear microscopy will likely miss a substantial fraction of potential source cases, although it is likely that these smear-negative, culture-positive cases are on average less infectious [24]. New rapid diagnostic tools with high sensitivity in HIV-infected inpatients, such as the GeneXpert test will play a pivotal role in identifying cases earlier and reducing the risk of nosocomial transmission. Additional approaches for infection control, including the adoption of improved administrative, environmental, and personal-protective measures are urgently needed to address this hazard.

No single invention is likely to be effective in eliminating the spread of TB in hospitals. However, a combination of mask use, reduced hospitalization time, increased outpatient therapy, improved ventilation, rapid drug testing, HIV treatment and tuberculosis isolation facilities will likely begin to address the challenge of nosocomial transmission [8]. While these changes will incur short-term costs and require changing patterns of care, we believe that the data presented here demonstrate the potentially severe consequences of failing to address the very real and present threat of nosocomial transmission of TB in South Africa.
